# Identification and Initial Characterization of Prophages in *Vibrio campbellii*

**DOI:** 10.1371/journal.pone.0156010

**Published:** 2016-05-23

**Authors:** Nicola Lorenz, Matthias Reiger, Mauricio Toro-Nahuelpan, Andreas Brachmann, Lisa Poettinger, Laure Plener, Jürgen Lassak, Kirsten Jung

**Affiliations:** 1 Munich Center for Integrated Protein Science (CiPSM) at the Department of Biology I, Microbiology, Ludwig-Maximilians-Universität München, Martinsried, Germany; 2 Department of Microbiology, University of Bayreuth, Bayreuth, Germany; 3 Department of Molecular Structural Biology, Max-Planck-Institute of Biochemistry, Martinsried, Germany; 4 Department of Biology I, Genetics, Ludwig-Maximilians-Universität München, Martinsried, Germany; Naval Research Laboratory, UNITED STATES

## Abstract

Phages are bacteria targeting viruses and represent the most abundant biological entities on earth. Marine environments are exceptionally rich in bacteriophages, harboring a total of 4x10^30^ viruses. Nevertheless, marine phages remain poorly characterized. Here we describe the identification of intact prophage sequences in the genome of the marine γ-proteobacterium *Vibrio campbellii* ATCC BAA-1116 (formerly known as *V*. *harveyi* ATCC BAA-1116), which presumably belong to the family of *Myoviridae*. One prophage was found on chromosome I and shows significant similarities to the previously identified phage ΦHAP-1. The second prophage region is located on chromosome II and is related to *Vibrio* phage kappa. Exposure of *V*. *campbellii* to mitomycin C induced the lytic cycle of two morphologically distinct phages and, as expected, extracellular DNA from induced cultures was found to be specifically enriched for the sequences previously identified as prophage regions. Heat stress (50°C, 30 min) was also found to induce phage release in *V*. *campbellii*. Notably, promoter activity of two representative phage genes indicated heterogeneous phage induction within the population.

## Introduction

*Vibrio campbellii* ATCC BAA-1116, formerly known as *Vibrio harveyi* ATCC BAA-1116 [1], is a versatile marine γ-proteobacterium. In addition to its pelagic lifestyle *Vibrio campbellii* is able to infect shrimp and fish and associates saprophytically with algae [2, 3]. *V*. *campbellii* is hypothesized to be responsible for the “milky sea effect” [4, 5]. This phenomenon of a blue glowing sea was first described by mariners of the 17^th^ century [6]. When *V*. *campbellii* colonizes the haptophyte *Phaeocystis*, the bacterium excretes so-called autoinducers (AIs), which ultimately lead to bioluminescence as a result of quorum sensing—the bacterial ability to determine population cell density via chemical communication [5]. The milky sea can cover up to 16,000 km^2^ of the ocean and the phenomenon can even be detected from space. Bioluminescence lasts from several hours to a few days and disappears as fast as it had appeared [6], perhaps as a result of the rapid breakdown of the bacterial community [5].

Most, if not all, bacterial species are targeted by viruses which could in principle trigger such a population collapse [7]. These so called bacteriophages are the most abundant entities in the biosphere [8]. To infect a bacterial cell, phages penetrate the bacterial plasma membrane and release genetic material into the cytoplasm, which then drives either a lytic or lysogenic life cycle [9]. In the lytic cycle, the host cellular machineries are hijacked for the production of new virus particles [10]. After transcription and replication of the phage DNA, the viral components are encapsulated to form complete phages. Ultimately each bacterial cell is filled with typically 100–200 viruses and undergoes lysis, thus releasing the phages for a new round of infection [9]. In contrast the lysogenic cycle does not result in an immediate replication and release of new phages. Instead, the injected viral DNA is integrated into the genome of the host and replicates as part of the bacterial chromosome. Such an integrated phage genome is called a prophage and normally does not interfere with the host’s life cycle. The prophages remain dormant until a lytic cycle is induced, typically by adverse conditions such as physical stresses (e.g., UV light) or chemical factors (e.g., mitomycin C) [11–13]. It is noteworthy that putative prophages can be identified in more than 70% of the available bacterial genomes [7]. Moreover, seawater is one of the richest natural sources for free viruses, as it contains up to 2.5x10^8^ virus particles per milliliter [14].

In this study we analyzed the *V*. *campbellii* genome using PHAST (**PHA**ge **S**earch **T**ool) [15] and identified two putative intact myoviridal prophage regions. One of the prophage sequences maps to chromosome I and encodes a ΦHAP-1-like phage [16]. The second phage resembles a kappa-like *Vibrio* phage and its sequence is inserted into chromosome II [17]. After treatment of *V*. *campbellii* with mitomycin C or exposure to higher temperatures, two distinct phages were found in transmission electron micrographs. Parallel deep sequencing and qRT-PCR analysis confirmed the association of the microscopically identified phages with the two myoviridal prophage regions. Finally, fluorescence microscopy studies of two reporter strains expressing a *gfp* promoter fusion with a representative phage gene indicated heterogeneous induction within the *V*. *campbellii* population.

## Results and Discussion

### Identification of *V*. *campbellii* ATCC BAA-1116 Prophages

**Fig 1:**
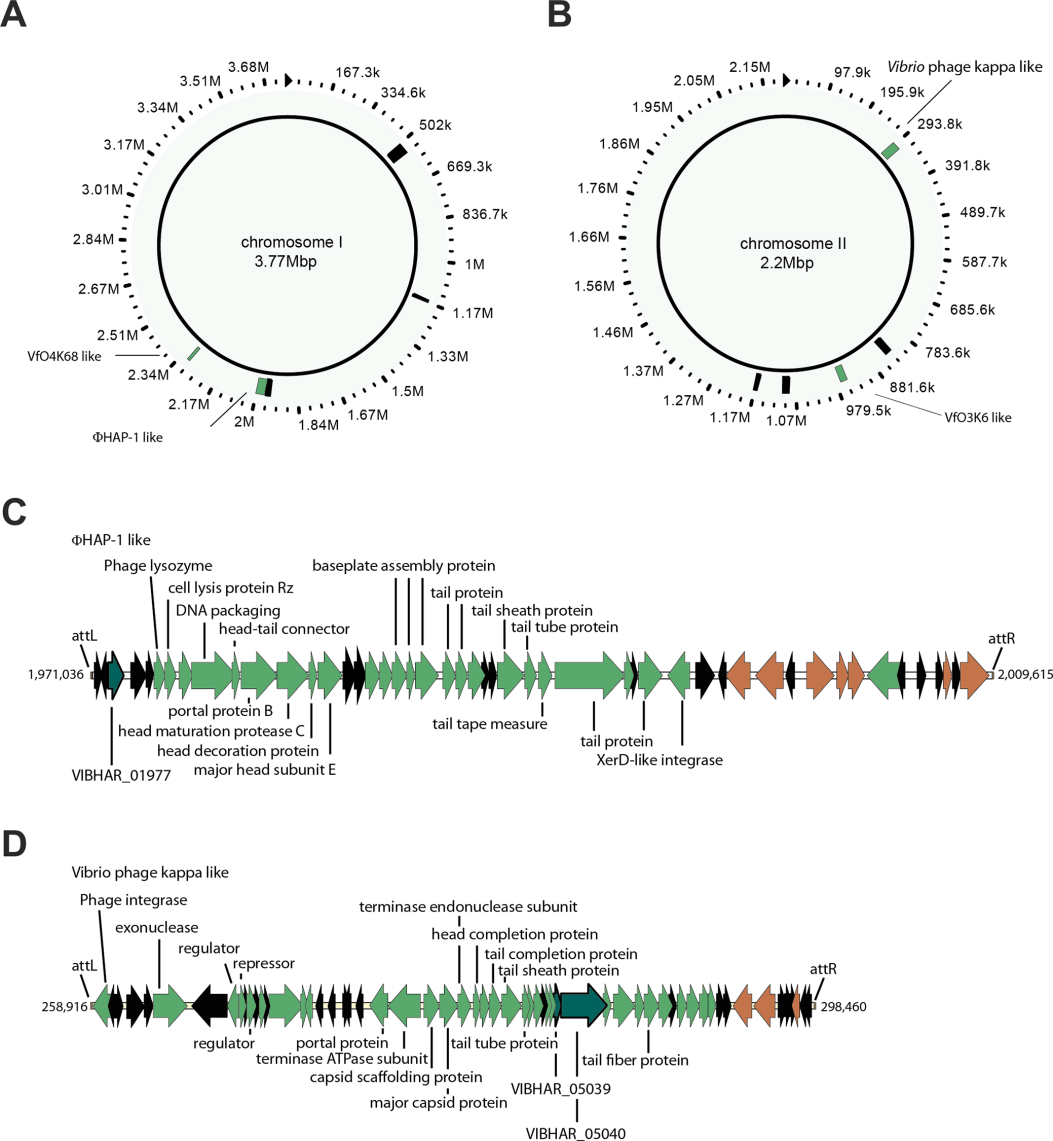
Bioinformatic identification of putative *V*. *campbellii* ATCC-BAA-1116 prophages. Putative prophage regions in *V*. *campbellii* ATCC BAA-1116 identified with PHAST [15]. **A**) *V*. *campbellii* ATCC-BAA-1116 chromosome I with five potential prophage regions indicated as green (probably intact) and black boxes (incomplete) **B**) Five potential prophage regions were also identified on chromosome II. Picture was adapted from the PHAST searching tool. **C**) Predicted organization of the putative ΦHAP-like prophage on chromosome I **D**) Predicted organization of the putative *Vibrio* kappa-like prophage on chromosome II; green: genes with annotated function in viral physiology; black: hypothetical genes; brown: transposase genes; dark cyan: genes selected for use in subsequent experiments.

We analyzed the genome of *V*. *campbellii* ATCC BAA-1116 for potential phage regions using PHAST [15]. On each of the two chromosomes five prophage regions, ranging in length from 12 kb to 45 kb, were detected (Fig 1A and 1B). Manual curation of the data however implied that in total only four of them are intact. Two of the apparently intact prophage regions are located on chromosome I. One of these prophages extends from bp 1,971,036 to 2,009,615 and is 38.5 kb in length (Fig 1A and 1C). Sequence analysis revealed significant similarities to the phage ΦHAP-1 (29.5% shared orthologous proteins) [16]. The second region encompasses bp 2,303,681 to 2,316,648 (13 kb) with similarities to *Vibrio* phage VfO4K68 (41.1% shared orthologous proteins) (Fig 1A) [18]. The other two intact prophages are inserted into chromosome II. Sequence comparison of the region between bp 258,916–298,460 (44.3 kb) suggests homologies to *Vibrio* phage kappa (28.8% shared orthologous proteins) (Fig 1B and 1D) [17]. The second prophage on chromosome II is related to *Vibrio* phage VfO3K6 (30.3% shared orthologous proteins) and is located between bp 961,672–968,454 (6.8 kb) [19].

The two prophages with similarities to VfO4K68 and VfO3K6, respectively, might be related to members of the genus *Inovirus*, whereas both the ΦHAP-1-like and kappa–like phages apparently belong to the family of *Myoviridae* [16–19]. Members of this family are typically characterized by an icosahedral capsid and a contractile tail. In agreement with our hypothesis, the identified genes are predicted to encode head and tail like structures (Fig 1C and 1D).

### Mitomycin C Treatment Induces the Lytic Cycle of *V*. *campbellii* Phages

In an attempt to stimulate prophage induction, exponentially growing *V*. *campbellii* cells were exposed to the cytotoxic antibiotic mitomycin C for 0.5 h (S1 Fig, time point I), washed twice and incubated further. After three hours, a significant drop in cell density was observed (S1 Fig) and might be indicative for cell lysis caused by phage release [20]. To check whether or not this was the case, we collected the supernatant and enriched the extracellular material for phage DNA isolation (S1 Fig, time point P). First, we performed a DNase I digestion to selectively degrade free extracellular DNA, leaving genetic information encapsulated in phage particles intact. We then subjected the supernatant to protease treatment to disrupt phage particles and release the viral DNA, which subsequently served as template for the generation of a Nextera XT (Illumina) library for MiSeq (Illumina) next generation sequencing. We obtained 2x300 bp paired-end sequences in more than 1,000,000 reads which could be mapped to the *V*. *campbellii* ATTC BAA-1116 reference genome. Genome coverage was markedly unequal, with sequences derived from the previously identified intact prophage regions being overrepresented in the enriched DNA fraction compared to extracellular chromosomal DNA. Coverage of the ΦHAP-1-like phage region on chromosome I at position 1,971,036–2,009,615 was more than twice the mean coverage of the chromosome (26.27 vs. 12.05) (S1 Table). Similarly, reads for the *Vibrio* kappa-like phage (position 258,916–298,460) were enriched (1.2 fold). Reads that mapped to the predicted prophage regions for the two putative Inoviruses, VfO4K68-like and VfO3K6-like, also enriched by a factor of 1.43 and 1.8, respectively. These results therefore suggest that treatment with mitomycin C indeed induces all of the intact prophages present in *V*. *campbellii*.

We also performed a sequencing run using exponentially grown cells of *V*. *campbellii* ATCC BAA-1116 that were not induced by mitomycin. We likewise found DNA enriched in the regions matching ΦHAP-1, VfO4K68, *Vibrio* phage kappa and *Vibrio* phage VfO3K6. This finding is not surprising as spontaneous phage induction was also reported for other bacteria such as *Shewanella oneidensis* and *Corynebacterium glutamicum* [21, 22].

Both DNA-sequencings (from Mitomycin induced and not induced *V*. *campellii)* revealed a relatively low enrichment of phage DNA compared to chromosomal DNA. This can be explained by the fact that phage independent cell lysis results in a release of bacterial DNA. In addition, the digestion steps (see Material and Methods section) influence the ratio. We have chosen very mild conditions to get as much phage DNA as possible, which in turn resulted in only partial digestion of bacterial DNA and a relatively low but significant fold change.

**Fig 2:**
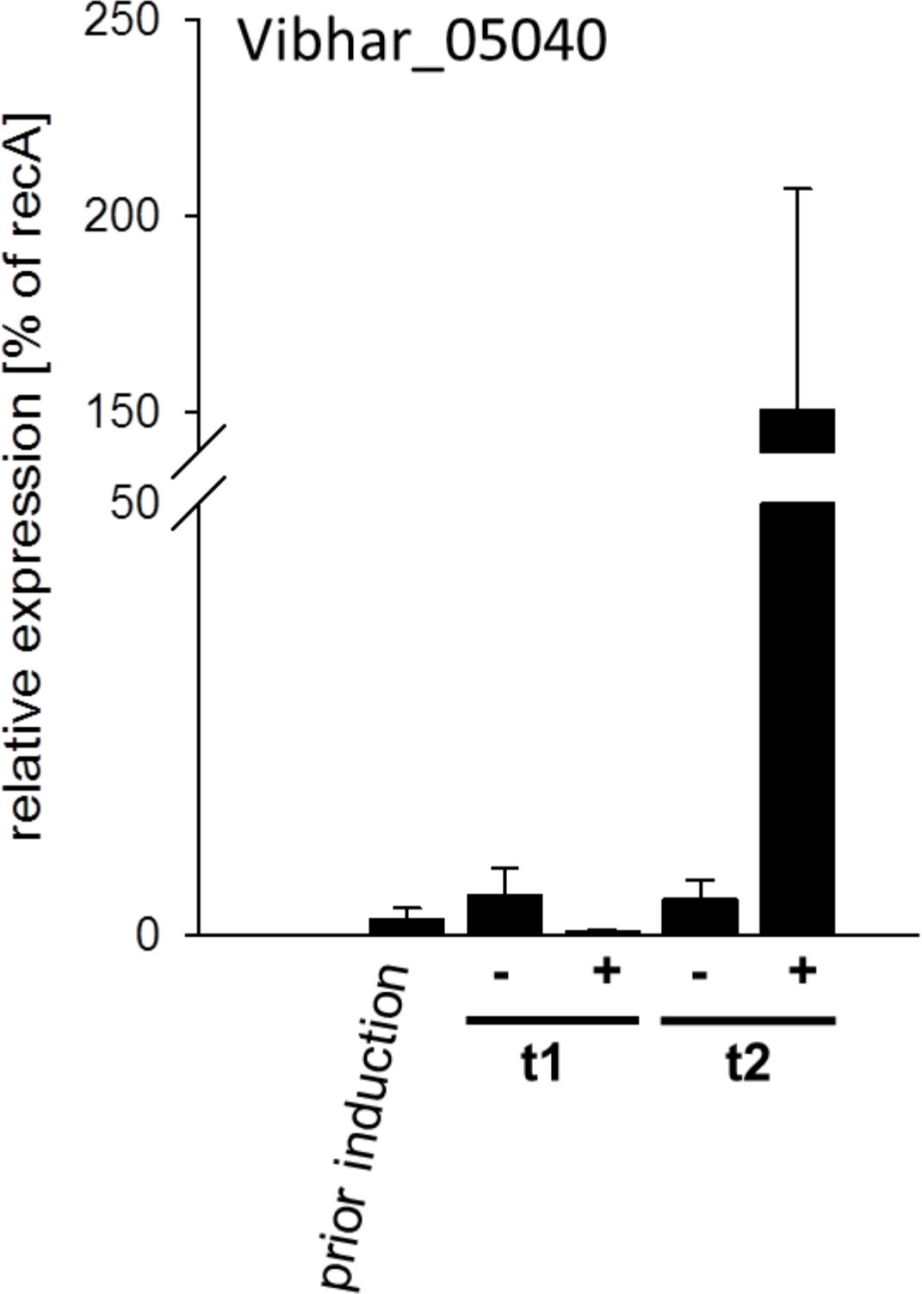
Mitomycin C induced transcriptional activation of the kappa-like *Vibrio* phage. Transcriptional analysis of Vibhar_05040 expression. *V*. *campbellii* ATTC BAA-1116 cells were grown to mid-exponential in AB medium at 30°C. After induction with mitomycin C for 0.5 h cells were washed twice with fresh AB medium and further incubated for 0.5 and 1.5 h. Samples for RNA extraction were taken prior to induction as well as 0.5 h (t1) and 1.5 h (t2) after induction. Levels of transcripts were determined by qRT-PCR for each time point. Changes in transcript levels (relative to *recA*) were calculated using the C_T_ method. All experiments were performed in triplicates and error bars represent the standard deviations of the mean. (+) induction with 1 μg/ml mitomycin C (-) DMSO control.

To further confirm our DNA sequencing results, we tested the viral gene expression by using qRT-PCR. We selected the least enriched prophage region of the *Vibrio* kappa-like phage and analyzed levels of Vibhar_05040 (Fig 1D) transcripts at various times after mitomycin C treatment. We observed a 30-fold increase of Vibhar_05040 transcript levels 1.5 hours after mitomycin C treatment compared to the DMSO treated control culture (Fig 2). These data are in agreement with the sequencing results and provide further evidence for the induction of prophages by chemical treatment in *V*. *campbellii*.

**Fig 3:**
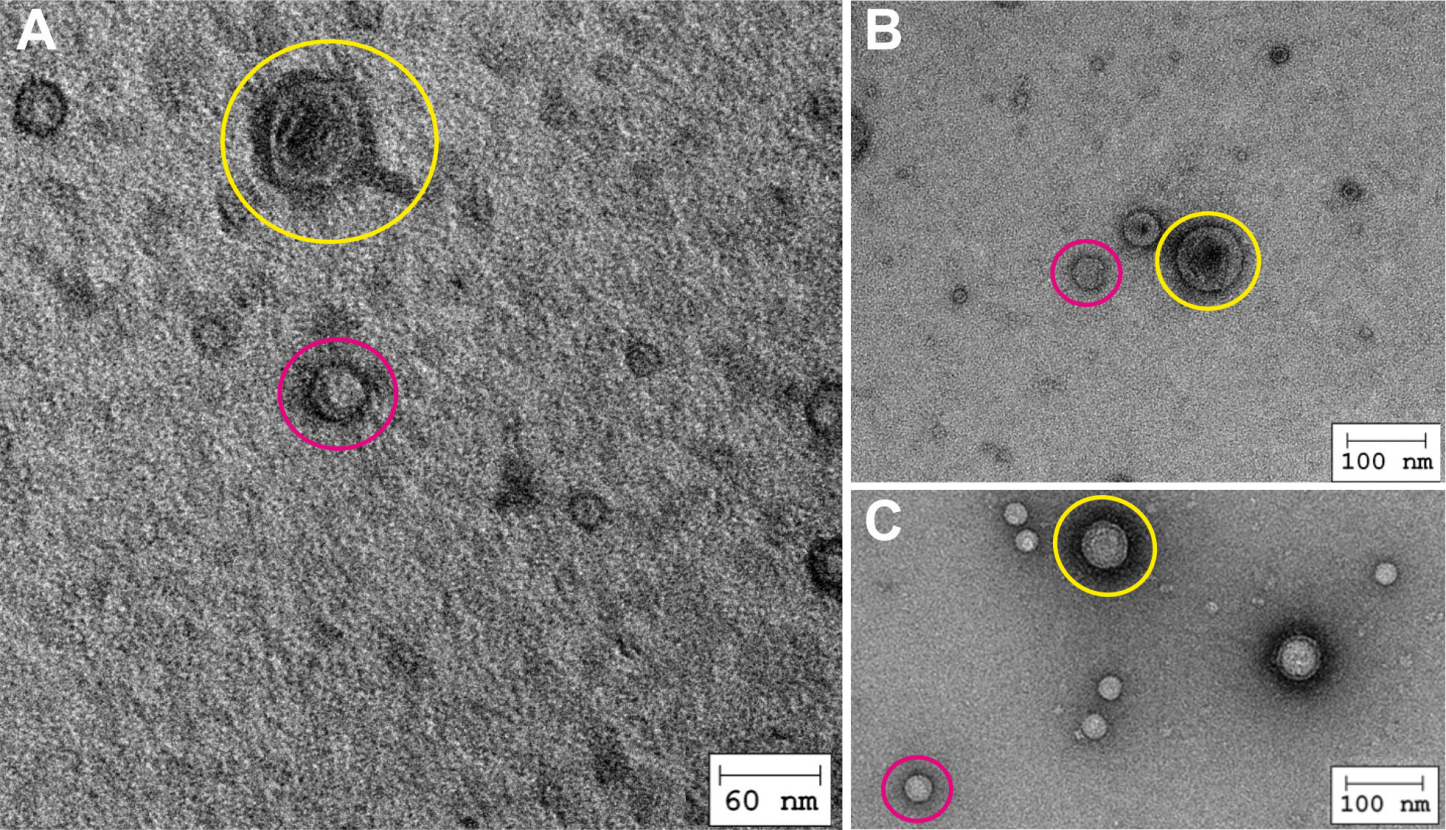
Visualization of two distinct types of icosahedral capsid particles. **A**) and **B**) TEM images of the lysate of *V*. *campbellii* ATTC BAA-1116 induced with 1 μg/ml mitomycin C revealed the presence of two phages of different sizes (yellow circle: d ~ 70 nm; pink circle: d ~ 45 nm). **C)** TEM images of the lysate of *V*. *campbellii* ATTC BAA-1116 induced after heat treatment at 50°C for 0.5 h revealed two phages of different sizes (yellow and pink circles). Scale bars are indicated.

Finally, we examined cell lysates using transmission electron microscopy (TEM) and detected two types of icosahedral capsid particles. However, none of the filamentous structures expected for the two putative Inoviruses were found (Fig 3). We believe that the number of virions, which were released from the bacteria, was too small to be detectable. Accordingly, the two capsid types most probably correspond to the two *Myoviridae* we identified on chromosome I and II, respectively. Although morphologically comparable, they differed significantly in size. The bigger capsid had a diameter of about 70 nm (Fig 3A and 3B, yellow circles), while that of the smaller was only about 45 nm (Fig 3A and 3B, pink circles). This in turn suggests differences in the genome size of these phages, which correlates with the putative genome sizes of 38.5 kb and 44.3 kb from the ΦHAP-1- and *Vibrio* kappa-like phages, respectively [23]. In addition to the capsid we also saw viral tails, but only for the larger phage particles (Fig 3A).

Taken together these results provide compelling evidence that the *V*. *campbellii* genome contains at least two intact prophages belonging presumably to the *Myoviridae* family that are shifted into the lytic cycle upon mitomycin C treatment.

Having a phage lysate in hand we asked whether we can reinfect *V*. *campbellii* ATCC BAA-1116 employing a classical plaque assay. However, we could not observe any plaque formation. This is not surprising as the incorporated prophages might protect *V*. *campbellii* from superinfection [21, 24]. Further studies on prophage free *Vibrio* strains are needed to investigate the infection potential.

### Heat Treatment Leads to the Release of Phages

We wondered whether other stress conditions might also trigger phage release. In their marine environment *V*. *campbellii* can often be found close to the surface and is therefore exposed to significant changes in temperature. Accordingly, an exponentially growing *V*. *campbellii* culture was exposed to heat (50°C for 0.5 h) and then shifted back to 30°C for 0.5 h. Subsequently, concentrated phage lysates were prepared and different dilutions of the concentrates were investigated via TEM. As in the case of mitomycin C treatment, two size classes of capsids could be distinguished (Fig 3C). Thus we speculate that, in their natural habitat phages can be released from *V*. *campbellii* by temperature shifts, and this might have an effect on behaviors such as quorum sensing by decreasing the population density.

### *V*. *campbellii* Phage Induction Is Heterogeneous

It has been reported that environmental stresses result in phage release from only a fraction of the bacterial population, with some phages remaining dormant [25]. To investigate the induction dynamics of the two intact *V*. *campbellii* phages, we analyzed viral promoter activities at the single cell level. For this purpose we constructed fluorescent reporter strains by fusing the promoter regions of Vibhar_01977 (part of the ΦHAP-1-like phage region) and Vibhar_05039 (part of the *Vibrio* phage kappa-like region), respectively (Fig 1C and 1D), to *gfp* and introduced them into the genome of *V*. *campbellii* via single homologous recombination at the native locus. The gene Vibhar_01977 codes for a potential DNA packaging protein and presumably plays a role in the assembly of the phage while Vibhar_05039 might have a role in tail assembly or exonuclease activity.

**Fig 4:**
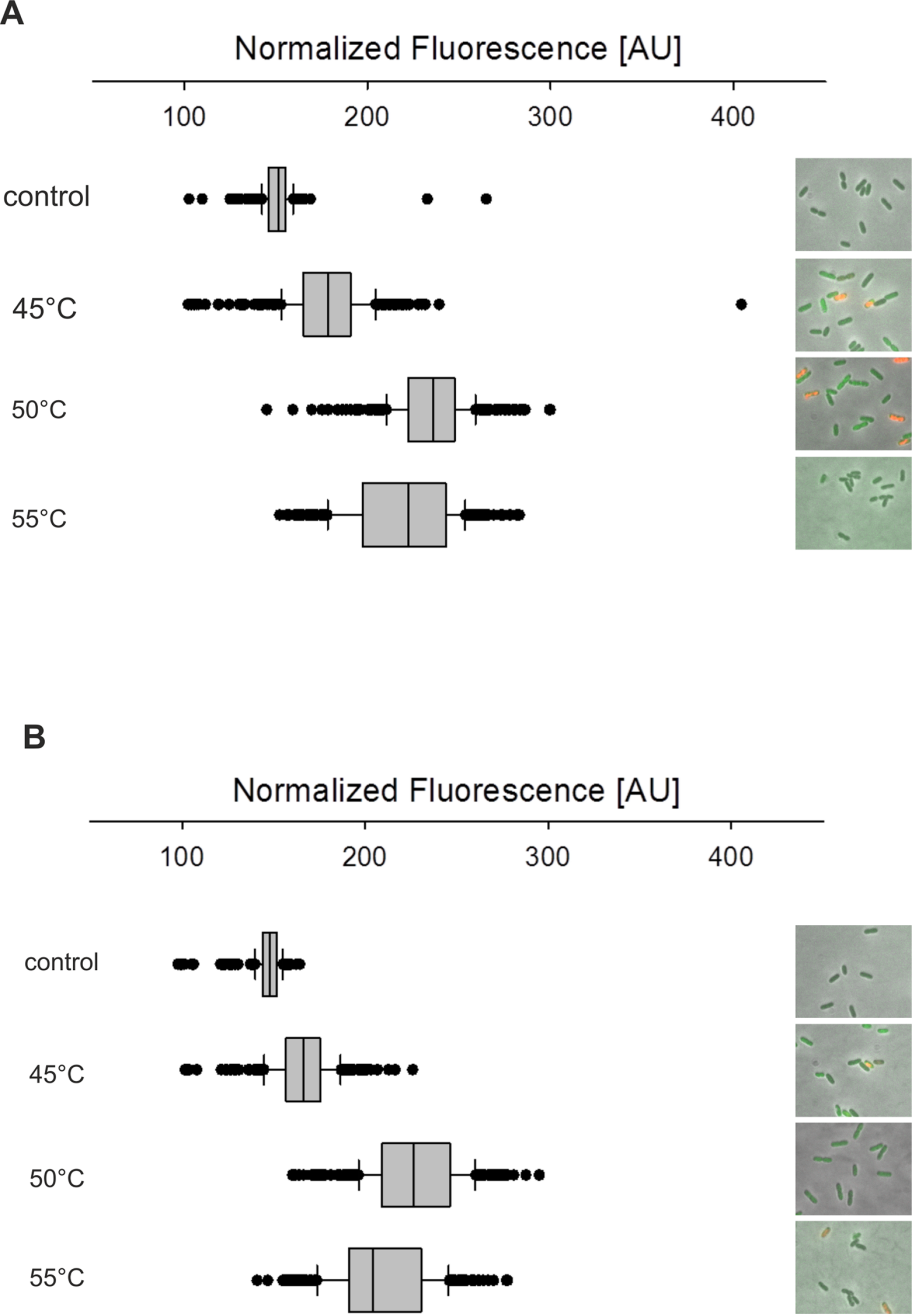
Population dynamics of *Vibrio* kappa- and ΦHAP-1-like phages. **A**) Single-cell microscopy imaging and GFP-fluorescence distribution in the *V*. *campbellii* ATTC BAA-1116 P_Vibhar-01977_-*gfp* population. In the exponential growth phase the culture was split and exposed to various temperatures (30, 45, 50, 55°C) for 15 min. Subsequently, cells were incubated for another 0.5 h at 30°C prior to microscopy. The box plots represent the distribution of GFP fluorescence signals from at least 350 cells. Red fluorescence in the pictures indicates dead cells after propidium iodide treatment. **B**) Same analysis as described in A) for *V*. *campbellii* ATTC BAA-1116 P_Vibhar-05039_-*gfp*. Images in A) and B) are representatives of three independent experiments.

To induce phage gene expression, we incubated *V*. *campbellii* cultures for 15 min at 30°C (control), 45°C, 50°C and 55°C and analyzed *gfp* reporter gene expression using fluorescence microscopy. The threshold for the determination of the OFF cells was set according to the mean fluorescence level observed in the 30°C control population (P_Vibhar_01977_-*gfp* = 170 AU and P_Vibhar_05039_-*gfp* = 160 AU; AU = arbitrary unit), which was comparable to a non-fluorescent *V*. *campbellii* wild type. An increase of temperature correlated with an increase in fluorescence intensity. A shift to 45°C resulted in activation of P_Vibhar_01977_ in about 66% of the population, increasing the mean fluorescence intensity slightly to 177 AU (Fig 4A). GFP production became even more pronounced when this reporter strain was incubated at 50°C and 55°C, resulting in an increase in the mean fluorescence intensity to 235 AU and 219 AU, respectively (Fig 4A). At those temperatures almost 100% of the cells became fluorescent. Concomitantly, the percentage of dead cells increased from about 1% in the control culture to 5–50% at higher temperatures. Vibhar_05039 promoter became activated in 71% of the cells after incubation at 45°C whereas 95–100% of the population were fluorescent at 50°C or 55°C. It is noteworthy that the fluorescence signal of individual cells differed in intensity, ranging from low (160/170-200 AU) to mid (200–250 AU) and high fluorescence (>250 AU) (Fig 4B).

The observed heterogeneity of phage induction at 45°C might be the result of a bet- hedging strategy [26], whereas the almost homogenous phage induction seen at higher temperatures would be expected to cause a population collapse due to simultaneous lysis of most of the cells.

## Conclusions

In the present study we report the identification of two intact prophages presumably belonging to the family of *Myoviridae*, which can be induced not only by mitomycin C but also by heat stress. While six lytic phages against different *Vibrio* strains including *V*. *campbellii* and *V*. *harveyi* were isolated in 2010 [27] this is to our knowledge the first report on prophage induction and analysis from *V*. *campbellii* ATCC BAA-1116.

Sequence homology to known phages suggests that the two phages might differ in their corresponding host range. Reportedly, the infection potential of the kappa-type phages seems to be restricted to the genus *Vibrio* [28, 29]. Accordingly one might speculate, that the kappa-like prophage in the *V*. *campbellii* genome is equally specific. On the contrary, the second myoviridial phage ΦHAP-1-like is related to a virus previously identified in *Halomonas aquamarina* [16]. Halomonads do not belong to the *Vibrionales* order of γ-proteobacteria but instead form a family within the *Oceanospirillales*, and with this indicating a wider host spectrum. We performed Blast Analysis for both of the two phages using at least two structural proteins as template sequence. For the ΦHAP-1-like virus we recognized homologies to certain phages in the genomes of several *Vibrio* species but also in Pseudomonads and Alteromonads such as *Shewanella* or *Halomonas* species. In case of the kappa-like prophage we found homologous proteins mainly distributed among Vibrios but also in certain Alteromonads. Thus we believe that the identified phages infect a broader range of γ-proteobacteria.

*Vibrio* species are among the most important bacterial pathogens in shrimp farms [30], prophages that can induce lysis of *V*. *campbellii* could serve as a basis for the development of a phage therapy in this setting. Bacteria and their phages interact in a multitude of ways. For instance, in *Shewanella oneidensis* MR-1, spontaneous phage induction during early growth phases is accompanied by the release of DNA, and is essential for proper biofilm formation [21]. A second example is the recently discovered phage induction by autoinducer-2 (AI-2) in *Enterococcus faecalis* [31]. AI-2 also mediates interspecies quorum sensing, and its association with phage release in *E*. *faecalis* facilitates the distribution of virulence genes via lateral gene transfer. Similarly, AI-2 is an integral part of the quorum sensing network in *V*. *campbellii* [32], and thus it is conceivable that phage mediated lysis of *V*. *campbellii* might also mediate bacterial population dynamics.

## Material and Methods

### Bacterial Strains and Growth Conditions

Strains and plasmids used in this study are listed in Table 1. The *E*. *coli* strains were aerobically grown in lysogeny broth (LB) (10 g/l NaCl, 10 g/l tryptone, 5 g/l yeast extract) at 37°C on a rotary shaker. The *V*. *campbellii* strains were cultivated in autoinducer bioassay (AB) medium [33] or Luria marine (LM) medium (20 g/l NaCl, 10 g/l tryptone, 5 g/l yeast extract) and were grown aerobically in a rotary shaker at 30°C. When required, media were solidified by using 1.5% (w/v) agar. If necessary, media were supplemented with 50 μg/ml kanamycin sulfate and/or 100 μg/ml ampicillin sodium salt. The conjugation strain *E*. *coli* WM3064 was grown in the presence of 300 μM *meso*-diaminopimelic acid (DAP).

**Table 1:** Bacterial strains and plasmids used in this study.

Strain or plasmid	Relevant genotype or description	Reference or source
**Bacterial strains**		
*Escherichia coli* DH5α-λpir	F^-^φ80d*lacZ* ΔM15 Δ (*lacZYA*-*argF*)U169 *rec*A1 *hsd*R17 *deoR thi*-1 *sup*E44 *gyr*A96 *rel*A1/λ*pir*	[41]
*Escherichia coli* WM3064	*thrB*1004 *pro thi rpsL hsdS lacZ* ΔM15 RP4-1360 Δ(*araBAD*)567 Δ*dapA*1341::[*erm pir*(wt)]	W. Metcalf, University of Illinois, Urbana-Champaign
*Vibrio campbellii* ATCC BAA-1116	wild type	[42]
*Vibrio campbellii* ATCC BAA-1116 P_Vibhar-01977_-*gfp*	Integration of P_Vibhar-01977_-*gfp* at the native locus in *Vibrio campbellii* ATCC BAA-1116	This study
*Vibrio campbellii* ATCC BAA-1116 P_Vibhar-05039_-*gfp*	Integration of P_Vibhar-05039_-*gfp* at the native locus in *Vibrio campbellii* ATCC BAA-1116	This study
**Plasmids**		
pNPTS138-R6KT	*mobRP4*^+^ *ori*-R6K *sacB;* suicide plasmid for in-frame deletions; *Km*^*r*^	[43]
pNPTS138-R6KT-GFP	*mobRP4*^+^ *ori*-R6K *sacB gfp; Km*^*r*^	This study
pNPTS138-R6KTP_Vibhar_01977_-*gfp*	*mobRP4+ ori*-R6KP_Vibhar_01977_:: *gfp*; Km^r^	This study
pNPTS138-R6KTP _Vibhar_05039_-*gfp*	*mobRP4+ ori*-R6KP_Vibhar_05039_:: *gfp; Km*^*r*^	This study

Induction of prophages was performed using mitomycin C or heat treatment of the culture. Mitomycin C (final concentration of 1 μg/ml) was added to the culture during the exponential growth phase for 30 min. Cells were then washed twice using fresh AB medium, further incubated and analyzed at designated time points. For heat treatment cells were exposed to 45°C, 50°C or 55°C for 30 min and afterwards shifted back to 30°C for subsequent analysis.

### Construction of Promoter Fusions

Molecular methods were carried out according to standard protocols [34] or according to manufacturer´s instructions. Kits for the isolation of plasmids and purification of PCR products were purchased from Südlabor (Gauting, Germany). Enzymes were purchased from New England Biolabs (Frankfurt, Germany) and Fermentas (St. Leon-Rot, Germany). Replicative plasmids were transferred into *E*. *coli* strains using chemically competent cells prepared as described in [35].

For construction of the promoter-*gfp* fusions (P_Vibhar_*01977*_ and P_Vibhar___*05039*_) 500 bp upstream of the coding sequence were amplified using the oligonucleotide pairs Vibhar_01977 500bp up BamHI s/Vibhar _01977 PspOMI as and Vibhar_05039 500bp up BamHI s/Vibhar_05039 PspOMI as, respectively (primer sequences can be found in S2 Table). The resulting promoter fragments were ligated into the γ-origin dependent vector pNPTS138-R6KT-GFP after restriction with BamHI and PspOMI.

Chromosomal insertions of promoter-*gfp* constructs into *V*. *campbellii* were achieved by integrating the resultant suicide vectors pNPTS138-R6KT-P_Vibhar_01977_-*gfp* and pNPTS138-R6KT-P_Vibhar_05039_-*gfp* via RecA dependent single homologous recombination as described previously [36]. The conjugative plasmid transfer from donor strain *E*. *coli* WM3064 containing the required plasmid into *V*. *campbellii* was performed as described above. Therefore, the donor and the recipient strain were cultivated in LB medium up to an OD_600_ of 0.8–1.0, supplemented with additives if required. Single colonies were checked for chromosomal integration via performance of a PCR with the genomic DNA.

### Single Cell Fluorescence Microscopy and Analysis

To measure promoter activity of P_Vibhar_01977_-*gfp* and P_Vibhar_05039_-*gfp* in individual cells, *V*. *campbellii* cells harboring the chromosomal fusions were cultivated in LM medium supplemented with 50 μg/ml kanamycin sulfate in a rotary shaker overnight. Cultures were then diluted 5,000 fold in AB medium and grown aerobically at 30°C. During exponential growth phase (OD_600_ 0.3–0.5) the culture was split equally into two flasks, one of which was shifted to 45°C, 50°C or 55°C for 15 min, while the second served as a control. Propidium iodide (Invitrogen, Oregon) was added to the cell suspension at a final concentration of 5 μM to stain dead cells (red fluorescence).

For phase contrast and fluorescence microscopy, samples were analyzed on 0.5% (w/v) agar pads, which were placed on microscope slides and covered by a coverslip. Images were taken on a Leica microscope (DMI 6000B) equipped with a Leica DFC 365 Fx camera. An excitation wavelength of 546 nm and a 605 nm emission filter with 75 nm bandwidth were used for visualization of dead cells. For GFP fluorescence, an excitation wavelength of 460 nm and a 512 nm emission filter with 75 nm bandwidth was used. For every condition three biological samples were analyzed and a minimum of 350 to 1,000 cells were evaluated for each parameter.

The obtained digital images were analyzed using the big Cell Brother software [37]. The software is open source and binaries are available at https://tmramalho.github.io/bigCellBrotherGUI.

### Preparation of Phage Concentrate

One liter of induced cells was centrifuged at 5,000 x g in a Sorvall Evolution centrifuge. The supernatant was filtered through a 0.2 μm filter to remove intact cells and cellular debris. The filtrate was then incubated with polyethylene glycol 6,000 (final concentration 100 g/l) overnight at 4°C, and the phage particles were pelleted by centrifugation in a Sorvall ultracentrifuge at 25,000 x g for 3 h. The pellet was resuspended in 4 ml SM buffer [10mM NaCl, 50 mM Tris/HCl (pH 7.5), 10 mM MgSO_4_] and incubated overnight at 4°C. The concentrate was filtered again and diluted for downstream applications.

### TEM

For conventional TEM analysis, 10 μl of phage concentrate (undiluted, 1:5 and 1:20 diluted) were adsorbed on glow discharged carbon coated copper grids (Plano, Wetzlar). After 10 min the phage solution was removed, and the sample was negatively stained by treatment for 30 s with 1% (w/v) uranyl acetate, briefly rinsed with a drop of deionized water, and then air dried. Bright-field TEM was performed on a FEI CM200 transmission electron microscope (FEI; Eindhoven, Netherlands) using an accelerating voltage of 160 kV. Images were captured with an Eagle 4k CCD camera using EMMenu 4.0 (Tietz) and FEI software.

### Extraction of Phage DNA and Sequencing

We used 170 μl phage concentrate and reduced non-viral DNA by adding 20 μl 10 Unit DNase I and 22 μl 10x DNase buffer. The mixture was incubated at 37°C for 3 h. To terminate DNase activity 25 μl of EDTA/SDS buffer (0.5 M EDTA, 1% (w/v) SDS) were added prior heating the phage concentrate to 65°C for 20 min. To remove the phage capsid structure 6 μl Proteinase K were added and the phage concentrate was further incubated at 65°C for 2 h with occasional shaking. After adding 300 μl chloroform and 75 μl 5M NaCl [38] the samples were transferred to PhaseTrap separation tubes (Peqlab, Erlangen). Aqueous phage DNA containing phase was separated from chloroform according to manufacturer’s instructions. Subsequently, DNA was precipitated and isolated according to Pospiech and Neumann [38].

For sequencing of phage DNA concentrates from strain *V*. *campbellii* ATCC BAA-1116 a library was generated with the Nextera XT Kit (Illumina) according to manufacturer's instructions. Sequencing was performed with a MiSeq sequencer (Illumina) yielding 2x300 bp paired-end sequences (v3 chemistry). For *V*. *campbellii* ATCC BAA-1116 1.1 x 10^6^ reads were obtained. Sequences were trimmed and assembled against the corresponding reference genomes of *V*. *campbellii* with CLC Genomics Server 6.0.5 (Qiagen) with the following parameters: mismatch cost = 2, linear gap cost, Insertion cost = 2, deletion cost = 3, insertion open cost = 6, insertion extend cost = 1, deletion open cost = 6, deletion extend cost = 1, length fraction = 0.5, similarity fraction = 0.8, non-specific match handling = map randomly. The coverage of the aligned reads to the reference genome was calculated using CLS Genomics Server 7.5 (Qiagen) with the following parameters: p-value threshold = 0.0001, minimum length = 50.

### Analysis of Transcription Levels via qRT-PCR

*V*. *campbellii* ATCC BAA-1116 was cultivated as described above. Samples were withdrawn, and RNA was isolated as described before [39]. The RNA was then used as template for random-primed first-strand cDNA synthesis according to the manufacturer’s instructions. Quantitative real-time PCR (qRT-PCR) (iQ5 real-time PCR detection system, Biorad) was performed using the synthesized cDNA, a SYBR-green detection system (Biorad) and specific internal primers for *recA* and Vibhar_05040 (S2 Table). The CT value (cycle threshold) was determined after 40 cycles using the iQ software (Biorad). Values were normalized with reference to *recA* and relative changes in transcript levels were calculated using the comparative CT method [40].

## Supporting Information

S1 Fig Effect of mitomycin C on growth of *V*. *campbellii*. Mitomycin C (final concentration of 1 μg/ml, indicated in green) and DMSO (control, indicated in red) was added to the culture during the exponential growth phase for 0.5 h (time point I). Then cells were washed twice in fresh AB medium. Phage lysate was prepared at time point P. Cell densities were determined by measuring optical densities at 600 nm (OD_600_).
(TIF)

S1 Table Relative mean coverage of sequencing reads of predicted *V*. *campbellii* ATCC BAA-1116 prophages. (PDF)

S2 Table Primers used in this study. (PDF)

## References

[1] Lin B, Wang Z, Malanoski AP, O'Grady EA, Wimpee CF, Vuddhakul V, et al Comparative genomic analyses identify the *Vibrio harveyi* genome sequenced strains BAA-1116 and HY01 as *Vibrio campbellii*. Environ Microbiol Rep. 2010;2(1):81–89. 10.1111/j.1758-2229.2009.00100.x 20686623 PMC2912166

[2] Jiravanichpaisal P, Miyazaki T, Limsuwan C. Histopathology, biochemistry, and pathogenicity of *Vibrio harveyi* infecting black tiger prawn *Penaeus monodon*. J Aquat Anim Health. 1994;6(1):27–35. 10.1577/1548-8667(1994)006<0027:HBAPOV>2.3.CO;2

[3] Catap ES, Lavilla-Pitogo CR, Maeno Y, Travina RD. Occurrence, histopathology and experimental transmission of hepatopancreatic parvovirus infection in *Penaeus monodon* postlarvae. Dis Aquat Organ. 2003;57(1–2):11–17. 10.3354/dao057011 14735916

[4] Widder EA. Bioluminescence in the ocean: origins of biological, chemical, and ecological diversity. Science. 2010;328(5979):704–708. 10.1126/science.1174269 20448176

[5] Lapota D, Galt C, Losee JR, Huddell HD, Orzech JK, Nealson KH. Observations and measurements of planktonic bioluminescence in and around a milky sea. J Exp Mar Biol Ecol. 1988;119(1):55–81. 10.1016/0022-0981(88)90152-9

[6] Miller SD, Haddock SH, Elvidge CD, Lee TF. Detection of a bioluminescent milky sea from space. Proc Natl Acad Sci USA. 2005;102(40):14181–14184. 10.1073/pnas.0507253102 16186481 PMC1242338

[7] Canchaya C, Proux C, Fournous G, Bruttin A, Brussow H. Prophage genomics. Microbiol Mol Biol Rev. 2003;67(2):238–276. 12794192 10.1128/MMBR.67.2.238-276.2003 PMC156470

[8] Ackermann HW. 5500 Phages examined in the electron microscope. Arch Virol. 2007;152(2):227–243. 10.1007/s00705-006-0849-1 17051420

[9] Ackermann HW, DuBow MS. Viruses of Prokaryotes: General properties of bacteriophages Boca Raton, FL: CRC Press; 1987.

[10] Campbell NA, Reece JB. Biology. Boston, MA: Pearson, Benjamin Cummings; 2005.

[11] Roberts JW, Devoret R. Lysogenic induction Cold Spring Harbor, NY Cold Spring Harbor Laboratory Press; 1983 123–144 p.

[12] Hershey AD, Dove W. Introduction to Lambda. Cold Spring Harbor, NY: Cold Spring Harbor Laboratory Press; 1983.

[13] Miller HI. Multilevel regulation of bacteriophage λ lysogeny by the *E*. *coli himA* gene. Cell. 1981;25(1):269–276. 10.1016/0092-8674(81)90252-X 6456071

[14] Bergh O, Borsheim KY, Bratbak G, Heldal M. High abundance of viruses found in aquatic environments. Nature. 1989;340(6233):467–468. 10.1038/340467a0 2755508

[15] Zhou Y, Liang Y, Lynch KH, Dennis JJ, Wishart DS. PHAST: A fast phage search tool. Nucleic Acids Res. 2011;39:W347–W352. 10.1093/nar/gkr485 21672955 PMC3125810

[16] Mobberley JM, Authement RN, Segall AM, Paul JH. The temperate marine phage ΦHAP-1 of *Halomonas aquamarina* possesses a linear plasmid-like prophage genome. J Virol. 2008;82(13):6618–6630. 10.1128/JVI.00140-08 18448537 PMC2447096

[17] Guidolin A, Manning PA. Genetics of *Vibrio cholerae* and its bacteriophages. Microbiol Rev. 1987;51(2):285–298. 3299030 10.1128/mr.51.2.285-298.1987 PMC373107

[18] Chang B, Miyamoto H, Taniguchi H, Yoshida S. Isolation and genetic characterization of a novel filamentous bacteriophage, a deleted form of phage f237, from a pandemic *Vibrio parahaemolyticus* O4:K68 strain. Microbiol Immunol. 2002;46(8):565–569. 10.1111/j.1348-0421.2002.tb02734.x 12363020

[19] Nasu H, Iida T, Sugahara T, Yamaichi Y, Park KS, Yokoyama K, et al A filamentous phage associated with recent pandemic *Vibrio parahaemolyticus* O3:K6 strains. J Clin Microbiol. 2000;38(6):2156–2161. 10834969 10.1128/jcm.38.6.2156-2161.2000 PMC86752

[20] Jiang SC, Paul JH. Significance of lysogeny in the marine environment: studies with isolates and a model of lysogenic phage production. Microb Ecol. 1998;35(3):235–243. 10.1007/s002489900079 9569281

[21] Gödeke J, Paul K, Lassak J, Thormann KM. Phage-induced lysis enhances biofilm formation in *Shewanella oneidensis* MR-1. ISME J. 2011;5(4):613–626. 10.1038/ismej.2010.153 20962878 PMC3105746

[22] Nanda AM, Heyer A, Kramer C, Grünberger A, Kohlheyer D, Frunzke J. Analysis of SOS-induced spontaneous prophage induction in *Corynebacterium glutamicum* at the single-cell level. J Bacteriol. 2014;196(1):180–188. 10.1128/jb.01018-13 24163339 PMC3911129

[23] Cui J, Schlub TE, Holmes EC. An allometric relationship between the genome length and virion volume of viruses. J Virol. 2014;88(11):6403–6410. 10.1128/jvi.00362-14 24672040 PMC4093846

[24] Bondy-Denomy J, Davidson AR. When a virus is not a parasite: the beneficial effects of prophages on bacterial fitness. J Microbiol. 2014;52(3):235–242. 10.1007/s12275-014-4083-3 24585054

[25] Nanda AM, Thormann K, Frunzke J. Impact of spontaneous prophage induction on the fitness of bacterial populations and host-microbe interactions. J Bacteriol. 2015;197(3):410–419. 10.1128/jb.02230-14 25404701 PMC4285972

[26] Veening JW, Smits WK, Kuipers OP. Bistability, epigenetics, and bet-hedging in bacteria. Annu Rev Microbiol. 2008;62:193–210. 10.1146/annurev.micro.62.081307.163002 18537474

[27] Crothers-Stomps C, Hoj L, Bourne DG, Hall MR, Owens L. Isolation of lytic bacteriophage against *Vibrio harveyi*. J Appl Microbiol. 2010;108(5):1744–1750. 10.1111/j.1365-2672.2009.04578.x 19886890

[28] Gerdes JC, Romig WR. Complete and defective bacteriophages of classical *Vibrio cholerae*: relationship to the kappa type bacteriophage. J Virol. 1975;15(5):1231–1238. 16789156 10.1128/jvi.15.5.1231-1238.1975 PMC354578

[29] Faruque SM, Bin Naser I, Fujihara K, Diraphat P, Chowdhury N, Kamruzzaman M, et al Genomic sequence and receptor for the *Vibrio cholerae* phage KSF-1phi: evolutionary divergence among filamentous vibriophages mediating lateral gene transfer. J Bacteriol. 2005;187(12):4095–4103. 10.1128/jb.187.12.4095-4103.2005 15937172 PMC1151723

[30] Defoirdt T, Sorgeloos P. Monitoring of *Vibrio harveyi* quorum sensing activity in real time during infection of brine shrimp larvae. ISME J. 2012;6(12):2314–2319. 10.1038/ismej.2012.58 22673627 PMC3504963

[31] Rossmann FS, Racek T, Wobser D, Puchalka J, Rabener EM, Reiger M, et al Phage-mediated dispersal of biofilm and distribution of bacterial virulence genes is induced by quorum sensing. PLoS Pathog. 2015;11(2):e1004653 10.1371/journal.ppat.1004653 25706310 PMC4338201

[32] Anetzberger C, Reiger M, Fekete A, Schell U, Stambrau N, Plener L, et al Autoinducers act as biological timers in *Vibrio harveyi*. PloS One. 2012;7(10):e48310 10.1371/journal.pone.0048310 23110227 PMC3482212

[33] Greenberg EP, Hastings JW, Ulitzur S. Induction of luciferase synthesis in *Beneckea harveyi* by other marine bacteria. Arch Microbiol. 1979;120(2):87–91. 10.1007/BF00409093

[34] Sambrook J. Molecular Cloning: A Laboratory Manual. Cold Spring Harbor, NY: Cold Spring Harbor Laboratory Press; 1989.

[35] Inoue H, Nojima H, Okayama H. High efficiency transformation of *Escherichia coli* with plasmids. Gene. 1990;96(1):23–28. 2265755 10.1016/0378-1119(90)90336-p

[36] Fried L, Lassak J, Jung K. A comprehensive toolbox for the rapid construction of *lacZ* fusion reporters. J Microbiol Methods. 2012;91(3):537–543. 10.1016/j.mimet.2012.09.023 23022912

[37] Plener L, Lorenz N, Reiger M, Ramalho T, Gerland U, Jung K. The phosphorylation flow of the *Vibrio harveyi* quorum sensing cascade determines levels of phenotypic heterogeneity in the population. J Bacteriol. 2015 10.1128/jb.02544-14 PMC4402392 25755191

[38] Pospiech A, Neumann B. A versatile quick-prep of genomic DNA from gram-positive bacteria. Trends Genet. 1995;11(6):217–218. 7638902 10.1016/s0168-9525(00)89052-6

[39] Fritz G, Koller C, Burdack K, Tetsch L, Haneburger I, Jung K, et al Induction kinetics of a conditional pH stress response system in *Escherichia coli*. J Mol Biol. 2009;393(2):272–286. 10.1016/j.jmb.2009.08.037 19703467

[40] Schmittgen TD, Livak KJ. Analyzing real-time PCR data by the comparative CT method. Nat Protocols. 2008;3(6):1101–1108. 10.1038/nprot.2008.73 18546601

[41] Miller VL, Mekalanos JJ. A novel suicide vector and its use in construction of insertion mutations: osmoregulation of outer membrane proteins and virulence determinants in *Vibrio cholerae* requires *toxR*. J Bacteriol. 1988;170(6):2575–2583. 2836362 10.1128/jb.170.6.2575-2583.1988 PMC211174

[42] Bassler BL, Greenberg EP, Stevens AM. Cross-species induction of luminescence in the quorum-sensing bacterium *Vibrio harveyi*. J Bacteriol. 1997;179(12):4043–4045. 9190823 10.1128/jb.179.12.4043-4045.1997 PMC179216

[43] Lassak J, Henche AL, Binnenkade L, Thormann KM. ArcS, the cognate sensor kinase in an atypical Arc system of *Shewanella oneidensis* MR-1. Appl Environ Microbiol. 2010;76(10):3263–3274. 10.1128/AEM.00512-10 20348304 PMC2869118

