# Supplementary figures and images for: Identification and Initial Characterization of Prophages in Vibrio campbellii
Source: PLoS One. 2016 May 23;11(5):e0156010. doi: 10.1371/journal.pone.0156010 (PMC4877103; doi:10.1371/journal.pone.0156010)

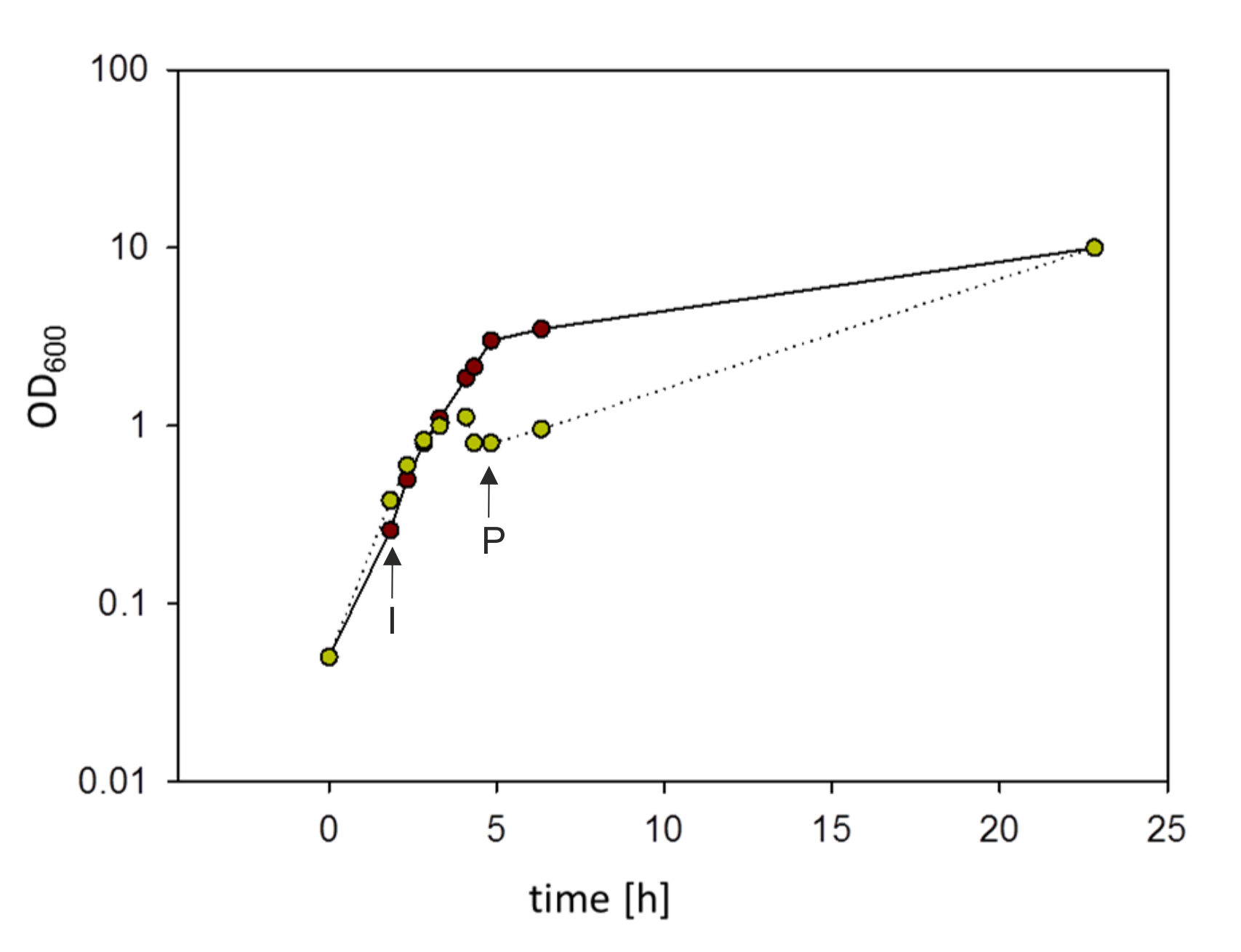

Supplement: S1 Fig — Mitomycin C (final concentration of 1 μg/ml, indicated in green) and DMSO (control, indicated in red) was added to the culture during the exponential growth phase for 0.5 h (time point I). Then cells were washed twice in fresh AB medium. Phage lysate was prepared at time point P. Cell densities were determined by measuring optical densities at 600 nm (OD600). (TIF) [file pone.0156010.s001.tif]
