# Supplementary material for: Identification and Initial Characterization of Prophages in Vibrio campbellii
Source: PLoS One. 2016 May 23;11(5):e0156010. doi: 10.1371/journal.pone.0156010 (PMC4877103; doi:10.1371/journal.pone.0156010)
Supplement: S1 Table — (PDF) [file pone.0156010.s002.pdf]

S1 Table

Chromosome	Region	Name	organism	mol_type	strain	Target region length	Target region length with coverage above 30	Percentage with coverage above 30	Read count	Base count	GC %	Min coverage	Max coverage	Mean coverage	Median coverage	Zero coverage bases	Mean coverage (excluding zero coverage)	Median coverage (excluding zero coverage)	Phage		
plasmid_NC_009777	1..89008	genomic DNA	Vibrio harveyi ATCC BAA-1116	genomic DNA	ATCC BAA-1116; BB120	89008	36746	41	21208	2263631	0.44	0	63	**25.43**	27	7440	27.75	28			
Chr1_NC_009783	1..3765351	chr I	Vibrio harveyi ATCC BAA-1116	genomic DNA	ATCC BAA-1116; BB120	3765351	36098	0	418682	45373306	0.46	0	155	**12.05**	12	235332	12.85	12			
Chr1_NC_009783	1971036..2009615	**pred-phageD intact**	Vibrio harveyi ATCC BAA-1116	genomic DNA	ATCC BAA-1116; BB120	38580	7541	19	8549	1013643	0.46	0	155	**26.27**	8	6013	31.13	9	phiHAP-1	Myoviridae	dsDNA
Chr1_NC_009783	2303681..2316648	**pred-phageE intact**	Vibrio harveyi ATCC BAA-1116	genomic DNA	ATCC BAA-1116; BB120	12968	236	1	2046	220772	0.42	1	32	**17.02**	17	0	17.02	17	VfO4K68	filamentous phage	DNA phage
Chr2_NC_009784	1..2204018	chr II	Vibrio harveyi ATCC BAA-1116	genomic DNA	ATCC BAA-1116; BB120	2204018	4217	0	221600	24011514	0.45	0	46	**10.89**	11	170021	11.81	12			
Chr2_NC_009784	258916..303231	pred-phageF intact	Vibrio harveyi ATCC BAA-1116	genomic DNA	ATCC BAA-1116; BB120	44316	1741	3	5199	561524	0.45	0	46	12.67	12	4090	13.96	13	Vibrio phage Kappa	Myoviridae	dsDNA
Chr2_NC_009784	961672..968454	**pred-phageI intact**	Vibrio harveyi ATCC BAA-1116	genomic DNA	ATCC BAA-1116; BB120	6783	502	7	1245	134020	0.46	6	42	**19.76**	19	0	19.76	19	Vibrio phage VfO3K6	Inovirus	ssDNA
