# Supplementary material for: Identification and Initial Characterization of Prophages in Vibrio campbellii
Source: PLoS One. 2016 May 23;11(5):e0156010. doi: 10.1371/journal.pone.0156010 (PMC4877103; doi:10.1371/journal.pone.0156010)
Supplement: S2 Table — (PDF) [file pone.0156010.s003.pdf]

S2 Table

Name	Sequence
M13 (-21) uni	TGTAAAACGACGGCCAGT
M13 (-29) rev	CAGGAAACAGCTATGACC
vibhar_05039 PspOMI as	CCGGGCCCTTTTTGCCTGTCGCTGCTCTC
vibhar_05039 500bp up BamHI s	CCGGATCCGAAACCGGACTCACCCGAATT
vibhar_01977 500bp up BamHI s	CCGGATCCTCGTTGAAAACAGTCGAGCTG
vibhar_01977 PspOMI as	CCGGGCCCTCATGGTTTTTGACCGGAAAC
05040_s	GCGTTGTTGCCTTCTCTCTTGCCTGT
05040_as	ATCATCAAGGTAAATGCACCGCCTGC
recA 240_s	GCTAACTCTTGAGCTTATTGCTGCTG
recA 395_as	AGCGCTTGCTCACCTGTGTCTGGC
